# Preserved Collateral Blood Flow in the Endovascular M2CAO Model Allows for Clinically Relevant Profiling of Injury Progression in Acute Ischemic Stroke

**DOI:** 10.1371/journal.pone.0169541

**Published:** 2017-01-09

**Authors:** Philip Little, Ola Kvist, Rikard Grankvist, Stefan Jonsson, Peter Damberg, Michael Söderman, Fabian Arnberg, Staffan Holmin

**Affiliations:** 1 Department of Clinical Neuroscience, Karolinska Institutet, Stockholm, Sweden; 2 Department of Neuroradiology, Karolinska University Hospital, Solna, Stockholm, Sweden; 3 Department of Radiology, Capio St Göran’s Hospital, Stockholm, Sweden; 4 Department of Materials Science and Engineering, Royal Institute of Technology, Stockholm, Sweden; 5 Karolinska Experimental Research and Imaging Center (KERIC), Karolinska University Hospital-Solna, Stockholm, Sweden; Henry Ford Health System, UNITED STATES

## Abstract

Interventional treatment regimens have increased the demand for accurate understanding of the progression of injury in acute ischemic stroke. However, conventional animal models severely inhibit collateral blood flow and mimic the malignant infarction profile not suitable for treatment. The aim of this study was to provide a clinically relevant profile of the emergence and course of ischemic injury in cases suitable for acute intervention, and was achieved by employing a M2 occlusion model (M2CAO) that more accurately simulates middle cerebral artery (MCA) occlusion in humans. Twenty-five Sprague-Dawley rats were subjected to Short (90 min), Intermediate (180 min) or Extended (600 min) transient M2CAO and examined longitudinally with interleaved diffusion-, T2- and arterial spin labeling perfusion-weighted magnetic resonance imaging before and after reperfusion. We identified a rapid emergence of cytotoxic edema within tissue regions undergoing infarction, progressing in several distinct phases in the form of subsequent moderation and then reversal at 230 min (p < 0.0001). We identified also the early emergence of vasogenic edema, which increased consistently before and after reperfusion (p < 0.0001). The perfusion of the penumbra correlated more strongly to the perfusion of adjacent tissue regions than did the perfusion of regions undergoing infarction (p = 0.0088). This was interpreted as an effect of preserved collateral blood flow during M2CAO. Accordingly, we observed only limited recruitment of penumbra regions to the infarction core. However, a gradual increase in infarction size was still occurring as late as 10 hours after M2CAO. Our results indicate that patients suffering MCA branch occlusion stand to benefit from interventional therapy for an extended time period after the emergence of ischemic injury.

## Introduction

Profiling the injury progression during acute ischemic stroke (AIS) has become increasingly relevant with the advent of pharmacological and endovascular treatment regimens. It is now understood that the rate of infarction progression is heterogeneous between AIS patients, and that slow infarction growth rates are associated with a higher potential to form a significant penumbra, and with improved clinical outcomes following recanalization treatment [1–3]. In cases of rapidly expanding malignant infarction [4–5], however, acute intervention is not appropriate, due to the unwarranted risk of adverse complications [6].

Experimental studies on injury progression during AIS have been limited by the use of animal models similar only to the malignant infarction profile. The commonly used intra-luminal suture occlusion model (MCAO) [7–8] causes a severe reduction of collateral blood flow through the circle of Willis and leptomeningeal anastomoses (LMAs), which rather than simulating typical cardio embolic or larger cortical or subcortical human AIS, produces extensive ischemic core regions mimicking the large hemispheric stroke found in patients with carotid-T occlusions [4, 9–10]. It is therefore warranted to examine the emergence and progression of ischemic injury in a model that simulates more closely the subtype of ischemic stroke that is suitable for acute intervention.

The M2 occlusion model (M2CAO) was designed specifically to simulate human stroke cases suitable for acute revascularization [11]. Selective M2 occlusion preserves collateral blood flow to the MCA from the anterior (ACA) and the posterior cerebral arteries (PCA), thereby producing ischemic lesions closer in relative size and with a regional blood flow situation similar to those commonly found in patients [10, 12]. The aim of this study was to use the M2CAO model to provide a clinically relevant profile of the emergence of ischemic injury in such cases.

Diffusion- (DWI) and T2 weighted (T2WI) magnetic resonance imaging (MRI) was used to identify tissue injury [13–16]. Regional perfusion weighted imaging (PWI) was used to assess the perfusion of tissue regions during M2CAO, and subsequently, to determine the spread of infarction at the expense of the ischemic penumbra, as defined by DWI/PWI mismatch [17–20].

## Materials and Methods

### Animal care and use

All animal handling and experiments, as well as humane endpoints, were conducted and applied according to the guidelines of the Animal Welfare Board at Karolinska Institutet and approved by the Stockholm Northern Regional Ethical Committee (N4/15). The care and use of animals in research is regulated by a common law frame across the EU (Directive 2010/63/EU). The Swedish legislation has been adjusted to the EU directive in 2013 and updated in 2015 (SJVFS 2015:24, L150). Experiments were conducted and reported in compliance with the Animal Research: Reporting in-Vivo Experiments (ARRIVE) guidelines. Animals were kept in groups in cages in a humidity controlled, thermoregulated facility with a 12 h/12 h light/dark cycle and access to food and water ad libitum. Animals were euthanized through decapitation while in deep anesthesia.

### Study design

**Fig 1:**
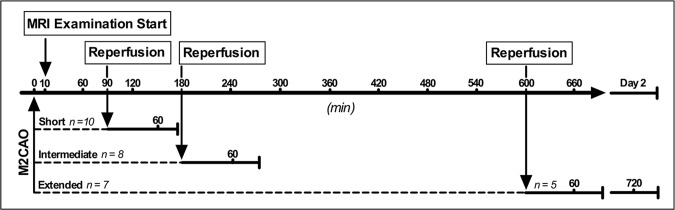
Study design outline. Three groups of animals were subjected to different durations of Middle Cerebral Artery M2 segment occlusion (M2CAO); Short 90 min M2CAO, Intermediate 180 min M2CAO, or Extended 600 min M2CAO. Animals were examined longitudinally with Magnetic Resonance Imaging (MRI) during M2CAO and after in-bore recanalization of the occluded vessel (reperfusion).

Twenty-five male Sprague-Dawley rats (Charles River, MA, USA) (weight mean ± SD; 400 ± 42.8 g) were included in the study. Animals were divided into three groups, subjected to either 90 min Short- (n = 10 animals), 180 min Intermediate- (n = 8) or 600 min Extended M2CAO (n = 7) (Fig 1).

Animals were transferred to, and placed within the MRI scanner within 10 min after the start of M2CAO. DWI and T2WI examinations were performed on all animals. PWI examinations were performed on all animals in the Intermediate and Extended M2CAO groups, and on 4 animals subjected to Short M2CAO. Imaging was performed in sequences, at 30 min intervals, with the initial examination at 30 min after the start of M2CAO. In a subgroup (n = 8), consisting of six animals from the Short M2CAO, the initial examination was performed earlier, at 10 min after the start of M2CAO, but no examination was performed at 90 min after M2CAO.

Subsequent to the assigned length of M2CAO, M2 recanalization (reperfusion) was performed in-bore. Reperfusion was followed by further MRI examinations, beginning within 30 min after reperfusion and continuing in 30 min intervals until the end of the imaging session on day 1.

Animals in the Short- and Intermediate M2CAO were examined only on day 1 and euthanized following its completion. Animals included in the Extended M2CAO group were returned to the MRI scanner the next day for a follow up examination at approximately 12 h after reperfusion. These animals were monitored for 2 h at the end of the examinations performed on day 1, and euthanized following examinations end on day 2.

### Surgical procedures

The M2CAO method is suitable for animals of 300–500 g body weight and was performed using a previously described procedure [11]. Anesthesia was induced at a 4%, and sustained at a 1.5–2%, Isoflurane (Virbac, Carros Cedex, France) concentration in an air: oxygen mixture (7:3). Animals were administered a 0,4 mL/kg dose of a local analgesic (Lidocain, 1%, Astra Zeneca, London, UK) before surgery. A heating pad with a rectal thermistor was used to maintain animal body temperature. Animal vital signs were monitored throughout the surgical procedure.

The endovascular procedure was performed under X-ray fluoroscopy guidance (Philips Allura Xper XD20 Interventional X-ray system, Philips Medical Systems). A 0.007-inch microwire (Hybrid; Balt Extrusion, Montmorency, France), sheeted inside a 0.020-inch microcatheter (Ultraflow; Covidien, Mansfield, MA, USA), was introduced through the ventral tail artery. The microcatheter was advanced to the proximal descending aorta, and subsequently, the tip of the microwire was navigated to, and positioned in, the M2 segment of the MCA, obtaining M2CAO. The surgical procedure from puncture to M2CAO took approximately 20–30 min per animal.

Reperfusion was achieved by retracting the microwire from the M2 segment and back into the microcatheter. Upon the transfer of animals for MRI, the proximal ends (farthest from the animal) of the microcatheter and microwire were left accessible outside the bore of the MRI-scanner, allowing minimally invasive reperfusion without disturbing the in-bore position of the animal.

### Acquisition and processing of magnetic resonance imaging

Animal vital signs were monitored continuously during in-vivo MRI imaging. A continuous flow of heated air, regulated by a rectal thermistor, was used to maintain animal body temperature during the examination. The head of the animal was placed in a stereotactic frame in order to maintain spatial consistency between examinations and to reduce motion artifacts.

MRI was performed in a horizontal 9.4 T magnet (Varian, Yarnton, United Kingdom) with a maximum gradient strength of 600 mT/m. An actively tuned birdcage resonator (Rapid Biomedical GmbH, Würzburg-Rimpar, Germany) and an actively detuned 4-channel phased array surface coil (Rapid Biomedical GmbH, Würzburg-Rimpar, Germany) were used for excitation and as receiving coil, respectively. An actively tuned arterial spin labelling (ASL) surface coil (Rapid Biomedical GmbH, Würzburg-Rimpar, Germany) was positioned under the neck of the animal. The coils were actively decoupled through a pin diode driver with three independent channels (Rapid Biomedical GmbH, Würzburg-Rimpar, Germany) in order to have only one coil tuned to the Larmor frequency at a time. Heart- and respiration rate and body temperature were monitored throughout the imaging session (SA-instruments, Stony Brook, NY, USA).

DWI and T2WI were acquired by multi-slice three-shot spin-echo echo-planar imaging at 13 slices of 1 mm thickness in the coronal plane. Repetition time (TR): 3 s, echo time (TE): 25 ms, field of view (FOV) of 32 x 32 mm, matrix size of 96 x 96 zero filled to 128 x 128 resulting in an in plane resolution of 256 μm. Diffusion gradient duration (Δ): 2.3 ms, diffusion gradient separation (δ): 6.5 ms. Diffusion sensitizing gradients were applied along 12 directions with a b value = 1000 s/mm^2^ and a control image collected twice with the diffusion sensitizing factor b = 0. Apparent diffusion coefficient (ADC) maps (mm^2^/s) were calculated from the collected data.

PWI was acquired by single-shot gradient echo planar imaging in combination with continuous ASL applying an off-resonance radio frequency power to the ASL coil concurrently with a 1 Gauss/cm gradient during TR. TR: 6 s, TE: 10.2 ms, FOV of 32 x 32 mm, matrix size of 64 x 64 zero filled to 128 x 128 resulting in an in place resolution of 256 μm. The labelling plane was located 2.4 cm upstream from the position of the center of the applied slice package, corresponding to 7 kHz off-resonance for the slice closest to the labelling plane, and increasing by 0.4 kHz for each consecutive slice. A set of tagged and non-tagged control images was acquired for each slice with 25 repetitions. The control image was acquired with the RF power of the tag coil set to zero. The localized excitation of the neck coil did not extend to the brain, and magnetization transfer could safely be neglected with the actively decoupled three-coil configuration. A slice package of identical configuration and position to that employed for DWI and T2WI was used. Cerebral blood flow (CBF) maps (ml/g/min) were calculated from the collected data. VnmrJ (Agilent Technologies, Palo Alto, CA, USA), ImageJ (National Institutes of Health, Maryland, USA), and OsiriX (OsiriX Foundation, Geneva, Switzerland) software were used for MRI data processing.

### Regions of interest

**Fig 2:**
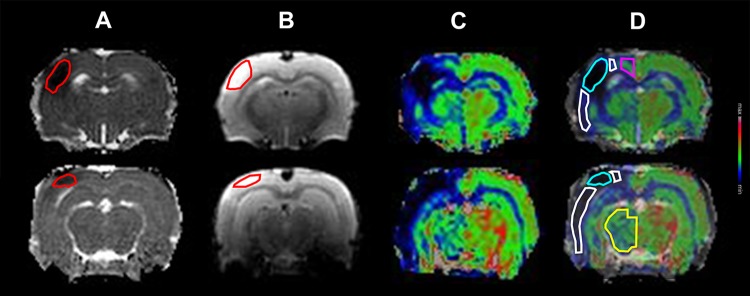
Regions of interest (ROIs). (A) Apparent diffusion coefficient (ADC) maps and (B) T2 weighted images with examples of ADC lesion and T2 lesion ROIs (red). (C) Cerebral blood flow (CBF) maps. (D) CBF maps with ADC overlay. ROIs used to examine the CBF of the Ischemic Core (cyan) were drawn initially in ADC maps at 60 min after reperfusion and subsequently transferred to CBF maps. ROIs delineating the Penumbra (white), regions supplied by the anterior cerebral artery (purple), and regions supplied by the posterior cerebral artery (yellow) were drawn in CBF maps.

To examine the progress of tissue injury within the ischemic lesion, for each of 25 animals and for every examination, individual regions of interest (ROIs) were traced manually around cortical volumes with decreased ADC, as well as around volumes of signal hyperintensity in T2 weighted images as seen in Fig 2A and Fig 2B respectively. At early time-points where no apparent T2 signal hyperintensity was discernable, the ROI from the first time point where T2 signal hyperintensity was detected was used.

To examine regional CBF, four subclasses of ROIs were employed within tissue regions. (1) ROIs delineating the Ischemic Core (Fig 2D), were traced manually over cortical regions with decreased ADC at 60 minutes after reperfusion and transferred to CBF maps calculated from PWI acquired at the initial examination (performed at 30 min M2CAO (n = 14), 60 min M2CAO (n = 1)). (2) ROIs delineating the peri-infarction Penumbra (Fig 2D), defined as hypoperfused tissue within the cortical regions supplied by the MCA, but not recruited into the final infarction. (3) ROIs delineating regions perfused by branches stemming from the ACA (ACAR) were traced manually across the supero-medial cerebral cortex (Fig 2D) at an average position extending from 3.85 ± 0.825 to -1.64 ± 1.82 mm relative to the bregma and with a mean volume of 18.8 ± 11.0 mm^3^. (4) For the PCA region (PCAR), ROIs were drawn over the thalamic, and part of the hypothalamic regions (Fig 2D) extending in average from -2.91 ± 0.573 to—4.91 ± 0.573 mm relative to the bregma, and at a mean volume of 37.2 ± 5.30 mm^3^. ImageJ and ITK-SNAP [21] software were used for ROI segmentation. During the application of ROIs, each examination was inspected visually inspected. There were a few instances when a discreet spatial shift was observed, and in those cases the placement of the ROIs was adjusted in order to maintain a consistent position of ROIs in the brain of animal.

### Image analysis

ADC values for each time point were normalized against a baseline ADC value acquired from a control region placed in the contralateral hemisphere in the first examination performed on the animal. A new baseline ADC control value, also collected from the contralateral hemisphere, was used for the 12 h follow up examination. In order to correct for local coil sensitivity inconsistencies, subsequent T2 lesion signal intensities were normalized against the ROI T2 lesion signal intensity from the image collected at the initial examination of that animal. T2 lesion signal intensities collected from the follow up examinations performed 12 h after reperfusion were normalized against a corresponding region in the contralateral hemisphere of the examined animal.

With the Ischemic Core, Penumbra, ACAR and PCAR ROIs, control regions of comparable sizes were drawn over corresponding tissue volumes in the contralateral hemisphere, and the full set of ROIs were subsequently applied to CBF maps calculated from scans performed at each consecutive time point. Absolute CBF values and CBF ratios were calculated for each region and each time point.

### Statistical methods

A regression analysis based on a linear mixed effects model was applied to the collected ADC and T2 signal intensity ratios from each examination. The analysis was set up as a one-way repeated measurements design with a first order autoregressive covariance structure. In order to analyze trends over time, we modelled the ADC and T2 –signal ratios as logarithmic and linear functions of time, and created a statistical model for each data set, consisting of linear and logarithmic trend components. The Akaike Information Criterion and residual analysis were used for model comparison and selection for each data set. F-tests were performed to test the statistical significance of individual trend components, with p < 0.05 considered significant.

Paired t-tests were used for analysis of regional CBF. Regional CBF in the ACAR and PCAR were subjected to retrospective co-variance analysis of all time points from 30–600 min M2CAO. Individual Spearman coefficients (ρ) were calculated for correlations between CBF ratios in the ACAR, PCAR and the CBF within the Ischemic Core and the Penumbra for each time point.

All statistical analyses were performed on group level, with the number of included animals specified in the results section. Statistical significance was defined as p < 0.05.

## Results

Two animals from the Extended M2CAO group suffered hemorrhagic transformation upon reperfusion. One of these animas died immediately and the other animal was euthanized, resulting in an 8% mortality rate throughout experiments. In these animals only imaging collected before reperfusion was included in the analysis. In two other animals (one animal from the Intermediate M2CAO group and one animal from the Extended M2CAO group), MRI scanner technical malfunction resulted in PWI data loss, and only the DWI and TW2I data was included in the analysis, leaving the number of animals where PWI could be used for longitudinal profiling of region specific CBF at 15 (Short group, n = 4 animals; Intermediate group, n = 7 animals, Extended group, n = 4 animals), and for ischemic lesion volume progression at 17 (Short group, n = 4 animals; Intermediate group, n = 7 animals, Extended group, n = 6 animals)

The initial examination during M2CAO was performed within 30 min after the start of M2CAO for all but one animal where it began after 60 min due to technical problems). The initial examination after reperfusion was likewise performed within 30 for all but one animal where it began after 60 min, also due to technical problems). Occasional failed examinations did occur during M2CAO and reperfusion. For a detailed presentation of all data points collected during MRI examinations, please see the supporting information.

### The emergence and progression of ischemic injury

**Fig 3:**
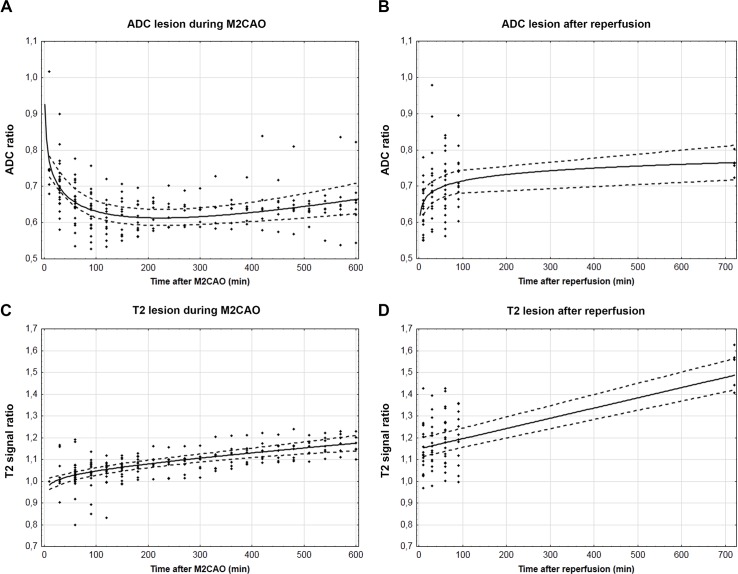
The emergence and progression of ischemic injury. Apparent diffusion coefficient (ADC) and T2 signal ratios between regions of interest and control regions (y-axes), measured at subsequent time points (x-axes) during M2 occlusion (A, C) and after reperfusion (B, D). Individual data points (diamonds), equation trend lines (solid lines), confidence intervals (dotted lines). Equations; A, y = 0.000328x – 0.0733log(x) + 0.93616, B, y = 0.0256log(x) +0.5967, C, y = 0.000180x + 0.02109 log(x) + 0.9317; D, y = 0.000470x + 1.14845.

**Fig 4:**
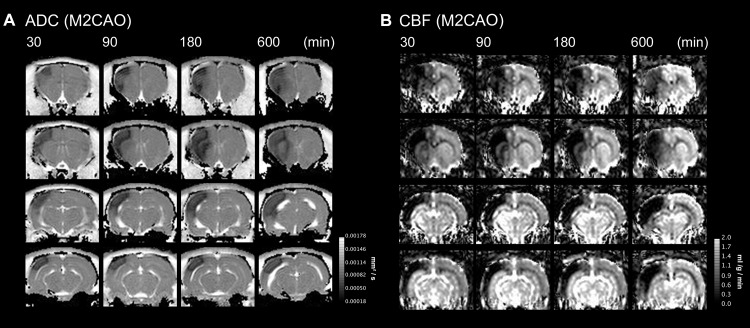
Tissue injury progression during occlusion of the M2 segment of the MCA (M2CAO). Apparent diffusion coefficient (ADC) maps (mm^2^/s) (A) and cerebral blood flow (CBF) maps (ml/g/min) (B), of four sections from a single animal at 30, 90, 180 and 600 min of M2CAO. At 30 min M2CAO, an ADC lesion is already present with diffusivity declining further while the volume increases at subsequent time points. The expansion of ADC lesion occurs at the expense of the region of diffusion / perfusion mismatch.

The ADC and T2 signal profile during M2CAO and during subsequent reperfusion can be found in Fig 3 (n = 25 animals). The initial, rapid decline of tissue ADC revealed an early emergence of cytotoxic edema within regions undergoing infarction, followed by a more moderate injury progression after the first 180 min of M2CAO (Fig 3A). A subsequent shift from ADC decrease in favor of a discreet normalization was detected at approximately 230 min after occlusion. The ADC profile during M2CAO of a representative animal is presented in Fig 4 with corresponding T2WI included as supplement in S3 Fig.

There were similarities between the emergence of cytotoxic and vasogenic edema, with the T2 signal in regions destined for infarction increasing at a higher rate in the beginning of the hyperacute phase, approximately over the initial 120 min of M2CAO (Fig 3C). The results from ADC and T2 signal intensity analyses were fitted with combined logarithmic and linear regression models, with both trend components significantly different from zero in both data sets (ADC: p values < 0.0001; T2 signal intensity: p values 0.0070 and 0.0032 respectively).

Upon reperfusion (n = 23 animals), we detected an increased rate of ADC normalization, to which was fitted a logarithmic trend line significantly different from zero (p value = 0.0002) (Fig 2B). T2 signal increase within the ischemic lesion also occurred at a comparatively higher rate after reperfusion (Fig 3D). The increase in T2 signal after reperfusion was fitted with a linear regression model significantly different from zero (p < 0.0001). None of the animals included in this study exhibited ischemic lesions within the ACAR or the PCAR.

### Regional blood flow of the Penumbra and of the Ischemic Core

**Fig 5:**
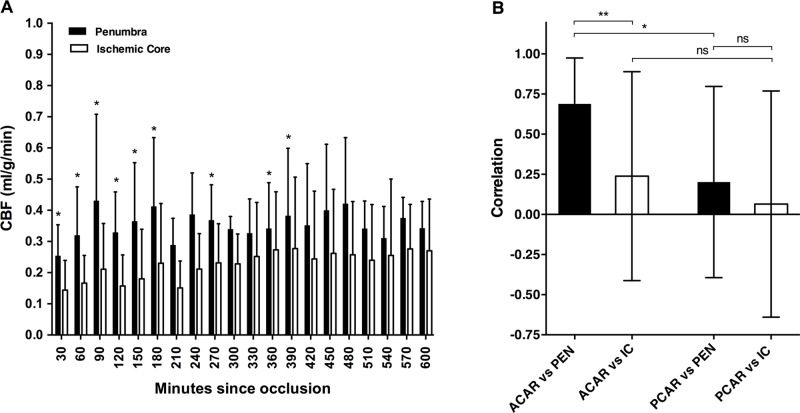
Cerebral blood flow during (CBF) during M2 segment occlusion (M2CAO). (A) CBF in the Ischemic Core and in the Penumbra. Results expressed as means and standard deviations (SD). *: p < 0.05, indicating a significant difference in CBF between the regions at the examined time point (In total 15 animas, Short Occlusion n = 4, Intermediate Occlusion n = 7, Extended Occlusion n = 4). (B) The correlation between the blood flow through tissue regions supplied by the anterior cerebral artery (leftmost bars) and regions supplied by the posterior cerebral artery (rightmost bars), and the Penumbra (PEN) and the Ischemic Core (IC), respectively. Mean and SD of Spearman r coefficients (y-axis) for the same 15 animals. Significant differences; ** p = 0.0088, * p = 0.0211.

M2CAO produces an early and apparent DWI / PWI mismatch, as can be seen in Fig 4. ROIs were subjected to longitudinal profiling of CBF (n = 15 animals). Although reduced, the blood flow of the Penumbra was significantly higher than in the Ischemic Core at all time points collected during the initial 180 minutes of occlusion (30, 60, 120, 150, 180 min: p < .0001; 90 min: p = 0.0006 (Fig 5A).

**Fig 6:**
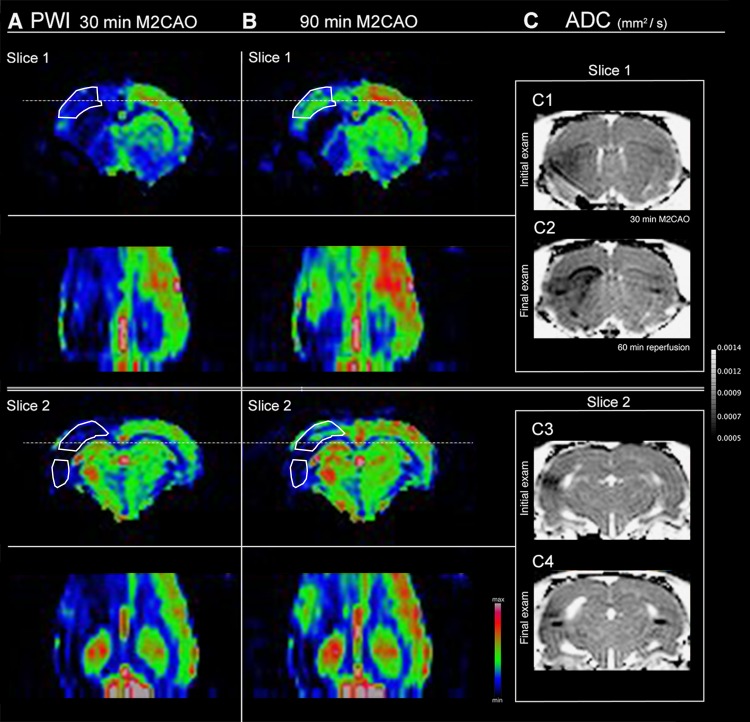
The correlation between blood flow in the Penumbra and blood flow in the anterior cerebral artery supply region (ACAR) during M2 segment occlusion (M2CAO). (A-B) Perfusion-weighted images (PWI) from a single animal at the initial examination at 30 min (A) and at the examination performed at 90 min (B) since the start of M2 occlusion (M2CAO), shown here in two slices, 1 and 2. Dotted lines show the levels of the coronal images. (A) There is hypoperfusion of the Penumbra (solid lines), as well as of the supero-medial cortical regions of the ACAR. (B) A substantial increase of ACAR perfusion in the supero-medial cortex occurs in tandem with an increase in blood flow through the Penumbra. C; Apparent diffusion coefficient (ADC) maps of Slice 1 (C1-2) and Slice 2 (C3-4), with C1, C3 showing the initial examination at 30 min M2CAO and in C2, C4 the final infarction at 60 min after reperfusion subsequent to Extended M2CAO (600 min). A localized image artifact resulting from the microwire positioned in the M2 can be seen in A-C.

In order to confirm the continued functionality of the leptomeningeal anastomotic network during M2CAO, we correlated the regional CBF of the Penumbra and the Ischemic Core to that of adjacent tissue regions not subject to ischemia. Our results revealed a significantly stronger CBF correlation between the ACAR and the Penumbra than between the ACAR and the Ischemic Core (p = 0.0088) (Fig 5B, representative example in Fig 6), as well as a significantly stronger CBF correlation between the ACAR and the Penumbra than between the PCAR and the Penumbra (p = 0.0211).

### The recruitment of Penumbra to the Ischemic Core

**Fig 7:**
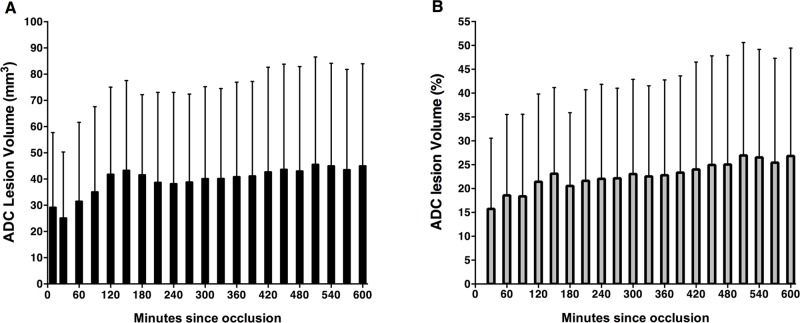
The recruitment of the penumbra to the ischemic core. (A) The progression of absolute growth of the ischemic lesion, defined by the decrease of the apparent diffusion coefficient (ADC), during M2 occlusion (M2CAO), presented as means and standard deviations (SD) for all 25 animals in the Short, Intermediate and Extended M2CAO groups. (B) The growth of the ADC lesion, presented as means and standard deviations of the percentage of the perfusion deficit volume at the initial examination in the Short (n = 4 animals), Intermediate (n = 7) and Extended M2CAO groups (n = 6).

The absolute growth of the ADC lesion was calculated for all time points during M2CAO (Fig 7A). The lesion grew from a mean ± SD of 25.2 ± 25.2 mm^3^ at 30 min, to 35.2 ± 32.5 mm^3^ (90 min), 41.7 ± 30.6 mm^3^ (180 min) to 45.1 ± 38.9 mm^3^ (600 min). All animals (n = 25) from the Short, Intermediate and Extended M2CAO groups were included in the analysis.

To determine the recruitment of Penumbra to the Ischemic Core during M2CAO, we determined the total volume of the initial perfusion deficit in the MCA territory. We subsequently calculated the fraction of that volume comprised of tissue undergoing infarction across time-points. The analysis included 17 animals from the Short (n = 4), Intermediate (n = 7) and Extended M2CAO (n = 6) groups. The ADC lesion grew from a mean ± SD of 15.7 ± 14.8% at 30 min, to 18.4 ± 17.2% (90 min), 20.5 ± 15.4% (180 min) to 26.9 ± 22.6% (600 min) (Fig 7B).

## Discussion

The revascularization of salvageable tissue may in best-case scenarios restore full health to AIS patients, providing that the therapeutic intervention occurs early enough, as *time is brain*. In order to proceed with an acute intervention, clinicians require reasonably accurate estimates of the degree of ischemic injury, as well as of the presence of potentially salvageable tissue. This is motivated by the fact that extensive injury prohibits acute treatment, as in the case of so-called malignant infarction, where a proximal occlusion results in a rapidly expanding ischemic core caused by the inhibition of collateral blood flow. The continuing focus on AIS associated disease processes in pre-clinical research is warranted by the potential to improve the diagnostic regimens currently used in clinical settings. The Intra-luminal MCAO model, although benefitting from high reliability and reproducibility, has major drawbacks relevant to the attempted simulation of human MCA branch occlusion; i.e. it produces ischemic lesions more akin to carotid T-occlusion including injuries to deep subcortical structure impacting the physiological homeostasis of the animal. Though deep subcortical injury can be avoided by using embolic stroke models, these do not allow for the controlled and immediate in-bore reperfusion required by our study design [9–10]. The aim of this study was accordingly to provide a detailed description of the progress of ischemic injury as modeled by M2CAO.

Our results outline an initial, rapid emergence of both cytotoxic and vasogenic edema in brain tissue undergoing infarction. The abrupt emergence of cytotoxic edema after M2CAO was replaced with a slow increase in ADC after 3 hours and 50 minutes of arterial occlusion, and subsequently by an ADC elevation following reperfusion. Several uncertainties surround the biological basis of these processes; in particular, whether elevation of ADC within the ischemic region represents actual tissue salvage or rather a pseudo-recovery resulting from large scale cell membrane rupture in ischemic cell populations, which would normalize the water diffusivity of the extracellular space [22–23]. The Reversal of ADC decline upon reperfusion following 10 min of ischemia in rats has previously been misinterpreted as signifying viability of affected tissue regions, with histological examination revealing widespread neuronal necrosis [24]. Accordingly, there are previous reports of regional tissue adenosine triphosphate (ATP) depletion at relative ADC levels of 77% of normal in rats, which would correspond to the ADC levels detected after approximately 10 min of occlusion (Fig 3A) in the present examination [25], indicating the early emergence of irreversible damage within the Ischemic Core. In contrast, vasogenic edema as identified by T2-signal elevation was not visually apparent until after 90–150 min of occlusion, but the rate of elevation did increase after reperfusion. This increased accumulation of vasogenic edema may be caused by blood brain barrier breakdown or reperfusion injury [15,26].

The relevance of vascular collateral profiling in AIS patients has increased with emerging reports of the impact on clinical outcome [27–31], with collateral status now indicated as a predictor of infarction expansion and of the risk of hemorrhagic transformation in patients undergoing endovascular recanalization [27, 30–31]. Previous studies describe a highly variable degree of ischemic lesion growth in early human AIS [1–3], with DWI lesion expansion occurring in around 70% of AIS cases and predominantly in patients with large perfusion mismatches in the acute phase [13, 32–33]. We report a longitudinal DWI/PWI mismatch profile of 11 percentage points of ADC lesion expansion relative to the size of the initially hypoperfused cortical tissue supplied by the MCA, with the volume fraction approximately 27% at 600 min MC2CAO (Fig 7B). Our results are congruent with data collected from stroke patients in whom lesion expansion during the acute and subacute disease phases occurred at the expense of DWI/PWI mismatch [1, 32, 34]; specifically, the DWI / PWI volume at 10 h after symptom onset has previously been documented at approximately 30%, followed by a further increase of the absolute lesion size as seen in follow up examinations performed 5 days after symptom onset [32]. We also show that perfusion of the ischemic Penumbra, not recruited to the final infarction, correlates significantly more strongly to the perfusion of the superior parasagittal cortex supplied by the ACA than does perfusion of regions undergoing infarction. We interpret the relative difference as an effect of collateral recruitment of the LMAs that provide connections between distal cortical branches of the large vessels of the brain [35]. This is in agreement with previous reports describing how the rapid dilation of LMAs following MCAO provides an increase in CBF to ischemic regions [36–37]. The results strongly indicate that collateral blood flow from adjacent tissue regions reaches the ischemic penumbra during M2CAO.

In conclusion, our results illustrate that even though the Ischemic Core rapidly undergoes infarction, the spread of infarction thereafter is slow, in all probability due to the preserved collateral blood flow of the M2CAO model. Interestingly, however, the infarction size in relation to the initial hypoperfused area continues to grow over a long period of time, which would favor an increased treatment window for revascularization therapy if an infarction-perfusion mismatch is detected. The present study contributes to the understanding of AIS pathophysiology, and provides a clinically relevant characterization of infarction evolution as modeled by M2CAO. Increased knowledge of ischemic injury progression has the potential to improve clinical decision making, and is necessary for the accurate evaluation of proposed neuroprotective and neurorestorative treatment strategies in the translational setting.

## Supporting Information

S1 Dataset Lesion ADC for all Animals during M2CAO and after Reperfusion. (XLSX)

S2 Dataset Lesion T2 Signal Intensity for all Animals during M2CAO and after Reperfusion. (XLSX)

S3 Dataset Region of interest CBF for all Animals during M2CAO and after Reperfusion. (XLSX)

S4 Dataset ADC Lesion Volumes and Initial hypoperfused MCA region Volume for all animals. (XLSX)

S1 Fig Lesion Apparent Diffusion Coefficient (ADC) Values on Group Level. Examinations performed during Short (n = 10 animals, A-B), Intermediate (n = 8, C-D) and Extended (n = 7, E; n = 5, F) middle cerebral artery M2 segment occlusion (M2CAo), and after reperfusion. Means (solid lines) and standard deviations (error bars) of performed examinations. **^**: < 3 animals examined at this time point. Control: ADCs of ipsilateral control regions collected at the initial examination for each animal.
(TIFF)

S2 Fig Lesion T2 Signal Intensity Ratios on Group Level. Examinations performed during Short (n = 10 animals, A-B), Intermediate (n = 8, C-D) and Extended (n = 7, E; n = 5, F) middle cerebral artery M2 segment occlusion (M2CAo), and after reperfusion. Means (solid lines) and standard deviations (error bars) of performed examinations. **^**: < 3 animals examined at this time point. Control: ADCs of ipsilateral control regions collected at the initial examination for each animal.
(TIFF)

S3 Fig Tissue injury progression in one animal during Extended occlusion of the M2 segment of the MCA (M2CAO). Apparent diffusion coefficient (ADC) maps (mm^2^/s) (A), T2 weighted images (B) and Cerebral blood flow maps (ml/g/min) (B), of five sections from a single animal at 30, 90, 180 and 600 min of M2CAO. At 30 min M2CAO, a small ADC deficit is already present with diffusivity declining further at subsequent time points. The expansion of the ADC lesion occurs at the expense of the region of diffusion / perfusion mismatch. T2-weighted signal elevation is not apparent initially, but increases throughout occlusion.
(TIF)
