# Supplementary figures and images for: Preserved Collateral Blood Flow in the Endovascular M2CAO Model Allows for Clinically Relevant Profiling of Injury Progression in Acute Ischemic Stroke
Source: PLoS One. 2017 Jan 9;12(1):e0169541. doi: 10.1371/journal.pone.0169541 (PMC5221807; doi:10.1371/journal.pone.0169541)

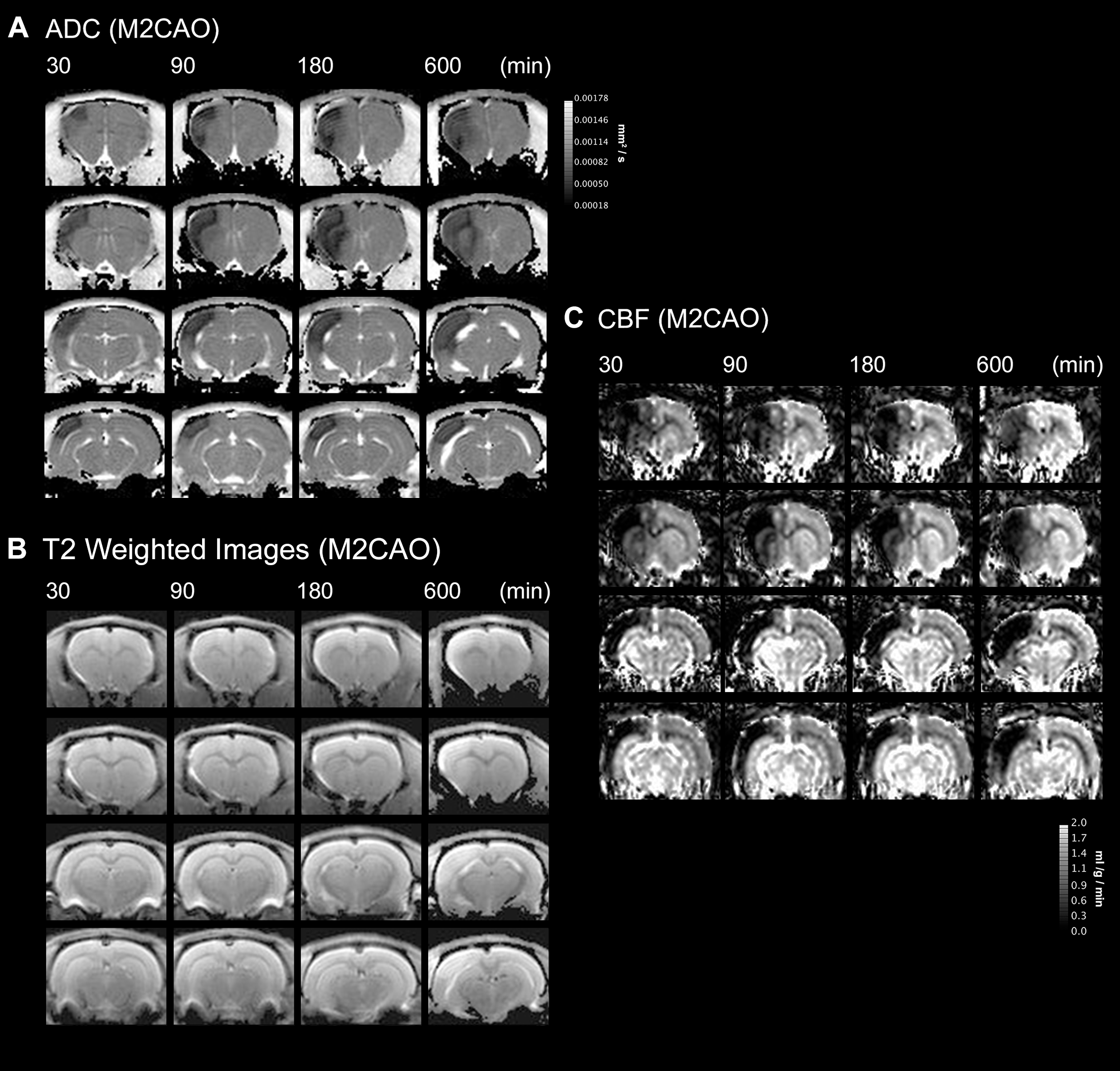

Supplement: S3 Fig — Apparent diffusion coefficient (ADC) maps (mm2/s) (A), T2 weighted images (B) and Cerebral blood flow maps (ml/g/min) (B), of five sections from a single animal at 30, 90, 180 and 600 min of M2CAO. At 30 min M2CAO, a small ADC deficit is already present with diffusivity declining further at subsequent time points. The expansion of the ADC lesion occurs at the expense of the region of diffusion / perfusion mismatch. T2-weighted signal elevation is not apparent initially, but increases throughout occlusion. (TIF) [file pone.0169541.s007.tif]
